# CpG increases vaccine antigen-specific cell-mediated immunity when administered with hepatitis B vaccine in HIV infection

**DOI:** 10.1186/1476-8518-6-4

**Published:** 2008-08-12

**Authors:** Jonathan B Angel, Curtis L Cooper, Jennifer Clinch, Charlene D Young, Andreane Chenier, Karl G Parato, Michael Lautru, Heather Davis, Donald W Cameron

**Affiliations:** 1 Ottawa Health Research Institute, 501 Smyth Rd., Ottawa, ON, K1H 8L6, Canada; 2 Division of Infectious Diseases, University of Ottawa, Ottawa Hospital – General Campus, 501 Smyth Rd., Ottawa, ON, K1H 8L6, Canada; 3 Coley Pharmaceuticals, 340 Terry Fox Dr., Suite 200, Ottawa, ON, K2K 3A2, Canada

## Abstract

**Background:**

Lack of adequate adjuvancy is a possible explanation for lack of vaccine immunogenecity. Immunostimulatory CpGs are potent vaccine adjuvants and may be an important component of the development vaccines, particularly those for which a cellular immune response is required for protection. We have previously demonstrated that CpG ODN co-administration with hepatitis B vaccine results in earlier, stronger and more sustained antibody responses to hepatitis B surface antigen in HIV infected individuals, and wished to determine if, in this population, helper T-cell responses were also enhanced.

**Methods:**

We conducted a double-blind, placebo-controlled trial in hepatitis B susceptible, effectively treated HIV-seropositive individuals. Participants received hepatitis B vaccine, with either placebo or CPG 7909 1.0 mg at week 0, 4 and 8. To determine the impact of CpG on cellular immune responses, lymphoproliferative responses (LPR) were evaluated by [^3^H]-thymidine incorporation at baseline and weeks 4, 8, 12, 24, and 48. Immunophenotyping of lymphocyte subsets was also determined at these time points.

**Results:**

Of 36 patients enrolled, 18 received hepatitis B vaccine alone, and 18 received hepatitis B vaccine with CpG. Inclusion of CPG 7909 was associated with a greater proliferative response to HBsAg at all time points following initial vaccination. This increase was statistically significant at 8 weeks (p = 0.042) and 48 weeks (p = 0.024). Similar results were observed when LPR were evaluated as stimulation indices (SI). No differences in proliferative responses to HIV p24 Ag were observed, nor were there any differences in lymphocyte subsets.

**Conclusion:**

In addition to enhancing humoral responses to vaccination, we describe for the first time that CPG 7909 enhances cellular immunity to vaccine antigen in a typically hyporesponsive population. This adjuvancy may be important in the development of an effective vaccine for which a cellular immune response is required for protection.

## Background

CpGs ODNs are immunostimulatory oligodeoxynucleotides that have recently gained considerable interest because of their ability to modulate the host immune response. By signaling through Toll-like receptor 9 (TLR9), CpG ODN preferentially induce type 1 (Th1) immune response, and therefore may be of value in the treatment of diseases that require T helper cell and cytotoxic T lymphocyte (CTL) responses for control of a specific pathogen or of a pathogenic immune process [1]. Also of interest, and a situation where a greater body of clinical data exists, is the potential use of CpG ODNs as vaccine adjuvants [2,1].

By improving the kinetics, magnitude and avidity of the antibody response, and the generation or augmentation of a cellular immune response (CD4+ T helper and CD8+ CTL responses) to vaccine, CpG ODNs have the potential to improve the quantity and quality of the vaccine-specific immune response [1].

CpG ODNs have been investigated extensively as adjuvants to a wide variety of antigens in numerous animal models. These have included pre-clinical studies of vaccines for both cancers, as well as a large number of infectious agents including influenza, hepatitis B virus (HBV), malaria, HIV, Herpes Simplex virus, tuberculosis, Leishmania, Toxoplasma, anthrax, tetanus, measles, hepatitis C virus and brucella, some of which have demonstrated that the inclusion of CpG improves protection from pathogen challenge [3-5].

In humans, CpG ODNs have been studied as adjuvants with various vaccines including influenza [6] and HBV [7,8]. Two different B-class CpG ODNs have been studied as an adjuvant to HBV vaccines in two separate Phase I studies with healthy volunteers. Both of these studies demonstrated that the inclusion of the CpG ODN resulted in the earlier appearance and a more sustained protective antibody response than the respective control vaccine [7,8].

In addition to enhancing antibody responses, CpG ODNs have been shown to induce or enhance cellular immune responses to HIV, toxoplasma, and HBV in mice [3,9-11]. Although there are limited data to suggest that CpG ODNs are capable of enhancing tumor specific T cell responses in human subjects with melanoma [12], there are no data published on the impact of CpG on the cellular immune response to vaccine "neo-antigen" when administered to humans.

The Phase I study testing CPG 7909 together with Engerix-B in healthy volunteers [7] led to a subsequent Phase Ib/II study of the same vaccine formulation in HIV-infected subjects [13]. The safety and immunogenicity aspects of that study have already been reported [13], and as in healthy volunteers [7], the addition of CPG 7909 was generally well tolerated and resulted in an earlier, stronger and more sustained antibody response. In this manuscript we report the effect of CPG 7909 on cell mediated responses in these subjects.

## Methods

### Study Design

Full details on the design of this phase Ib/IIa, study have previously been reported [13]. In brief, the study which included HIV+ subjects, aged 18–55 years was conducted at The Ottawa Hospital Clinical Investigation Unit, Ottawa, Canada. The study was approved by The Ottawa Hospital Research Ethics Board. Subjects were on highly active antiretroviral therapy (HAART) for a minimum of six months, with CD4 T lymphocyte counts ≥ 200 cells/μL and HIV RNA < 50 copies/mL for a minimum of three months. Subjects included in this component of the study all had anti-HBs titres <10 mIU/mL, half being vaccine naïve and half having failed a previous course of 3 or more doses with a commercial HBV vaccine. Subjects were anti-HBc, HBsAg and HBV DNA negative. These susceptible subjects were randomized to receive Engerix-B (GlaxoSmithKline, Rixensart BE) or Engerix-B admixed with CPG 7909.

### Vaccines and Control Injections

All subjects were dosed at 0, 1 and 2 months and received two intramuscular injections, one into each deltoid, of an adult dose of Engerix-B, thus a total of 40 μg HBsAg adsorbed to alum. Experimental vaccine recipients also received 0.5 mg CPG 7909 in 100 μl mixed with each injection of vaccine for a total dose of 1 mg CPG 7909. Control vaccine subjects received 100 μl of saline added to each vaccine injections. In all cases, the volume injected into each arm was 1.1 mL. CPG 7909, a B-Class CpG ODN of the human optimized sequence 5'-TCGTCGTTTTGTCGTTTTGTCGTT-3' was synthesized with a wholly phosphorothioate backbone (Coley Pharmaceutical Group, Wellesley MA).

### Immunological Evaluations

#### Lymphocyte proliferation assay (LPA)

To assess cell-mediated immune responses, whole blood was collected from all subjects at baseline and at 4, 8, 12, 24 and 48. Peripheral blood mononuclear clear cells (PBMC) were isolated from whole blood by Ficoll-Paque™ Plus *(*Pharmacia Fine Chemicals, Piscataway, NJ) gradient separation, washed twice in PBS and resuspended in RPMI medium 1640 (Invitrogen, Auckland, NZ) supplemented with penicillin-streptomycin (Invitrogen, Auckland, NZ) and 5% AB serum (Sigma, St. Louis, MO). PBMC were plated in triplicate at 3 × 10^5 ^cells/well and stimulated with various antigens. Vaccine antigen specific responses were assessed using recombinant yeast-derived HBsAg (2.5 mg/ml; subtype ad; International Enzymes Inc, Fallbrook, CA). Antigen used to assess HIV specific responses was HIV-1 gag p24 (5 mg/ml) (Immunodiagnostics, Inc, Woburn, MA). Mitogens, irrelevant antigens, and negative controls included: 1) pokeweed mitogen (PWM) (0.1 mg/ml; Sigma, St. Louis, MO), 2) tetanus toxoid (2 LFU/ml; Massachusetts Public Health Biologic Laboratories, Boston, MA), 3) Candida albicans antigen (10 mg/ml; Greer laboratories, Lenoir, NC), 4) cytomegalovirus (CMV), CF antigen strain AD169 (1:100; Biowhittaker, Walkersville, MD) 5) varicella-zoster virus (VZV) (1:100; Biowhittaker), 6) Keyhole limpet hemocyanin (KLH) (50 mg/ml; Sigma) and 7) no antigen. Cells were incubated for 6 days at 37°C prior to pulsing with ^3^H-thymidine (1 mCi/well) for a further 6 hours. Plates were harvested on glass fibre filters and counted in a 1450 microbeta Trilux scintillation counter (Wallac, Boston, Ma). Stimulation index (SI) was calculated by dividing the counts per minute (cpm) in the stimulated wells by the cpm from control unstimulated wells.

### Whole blood immunophenotyping

Peripheral blood samples were collected in a 5 ml vacutainer containing EDTA. Whole blood was incubated with BSA-Cy5 (Amersham Biosciences, Piscataway, NJ) for 10 minutes prior to staining with antibodies conjugated to fluorescein isothyocyanate (FITC), phycoerythrin (PE) or phycoerythrin-cyanin 5.1 (PC5) for 25 minutes in the dark. The antibodies specific for cell surface markers that were utilized were anti-CD3 (UCHT1), CD4 (13B8.2), CD8 (B9.11), HLA-DR (Immu-357), CD38 (T16), CD28 (CD28.2), CD45RA (ALB11), CD45RO (UCHL1), CD62L (DREG56), (all from Beckman coulter) and anti-CD95 (DX2) (BD Pharmingen, Mississauga, Canada). Lysing and fixing of cell preparations were performed using the ImmunoPrep™ reagent system in a Coulter Multi-Q Prep (Beckman-Coulter, Inc., Fullerton, CA). Ten thousand events were acquired on a Beckman Coulter ALTRA flow cytometer.

### Statistical Analysis

The number of patients in this study was chosen to identify important differences in measures of safety between subjects receiving CPG 7909 and those receiving placebo with their Engerix-B vaccines. Immunologic measures were summarized using group-wise means and 95% CI. Potential differences between the two treatment groups were evaluated using a repeated measures analysis of variance. The SAS mixed procedure was used with an autoregressive covariance structure. Since this method requires equal time intervals between measurements, two analyses were done for each dependent measure – one using weeks 4, 8 and 12 and a second using weeks 24 and 48. Three effects were modeled: treatment group, time and the treatment group by time interaction. The proportion of subjects with a positive proliferative responses (SI>5) in each group was compared using Chi square test.

## Results

Between January 2001 and August 2002, 38 subjects were enrolled into the two vaccine arms of this study, 19 of whom received Engerix-B plus CpG 7909 and 19 of whom received Engerix-B plus CpG plus saline. A complete description of patient characteristics was previously reported [13] but a brief summary is outlined in Table 1.

**Table 1:** Baseline Characteristics

	**Engerix-B** ** n = 19**	**Engerix B-CPG** ** N = 19**
Male (n)	18	14
Prior Vaccine Failure (n)	10	9
Age (Mean ± SD)	42.9 ± 7.3	41.0 ± 7.4
CD4+ T cell count (Mean ± SD)	543 ± 228	647 ± 262
Proportion with HIV RNA <50 copies/mL	100%	100%

Safety and some immunogenicity data have also been previously reported [13]. In brief, the groups of subjects receiving CPG 7909 with their HBV vaccine had a greater proportion of subjects achieving protective titers (≥ 10 MIU/ml), which were reached more quickly and were more sustained than in the control group of subjects. Specifically, seroprotective titres by 6 and 8 weeks, and 12 months were found in 89%, 89%, and 100% of subjects receiving CPG 7909 compared to 53%, 42%, and 63% of controls respectively (p = 0.029, 0.005, and 0.008) [13].

**Figure 1:**
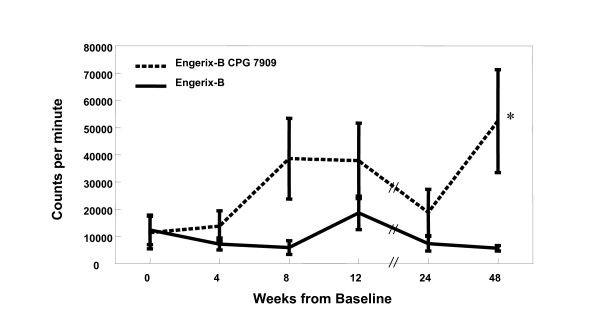
**Proliferative responses to hepatitis B surface antigen (HBsAg)**. Proliferative responses to HBsAg, measured as counts per minute (cpm) of ^3^H-thymidine incorporation. *Repeat measures ANOVA week 4, 8, 12, p = 0.0039, n = 38; week 24, 48, p = 0.02714, n = 38.

**Figure 2:**
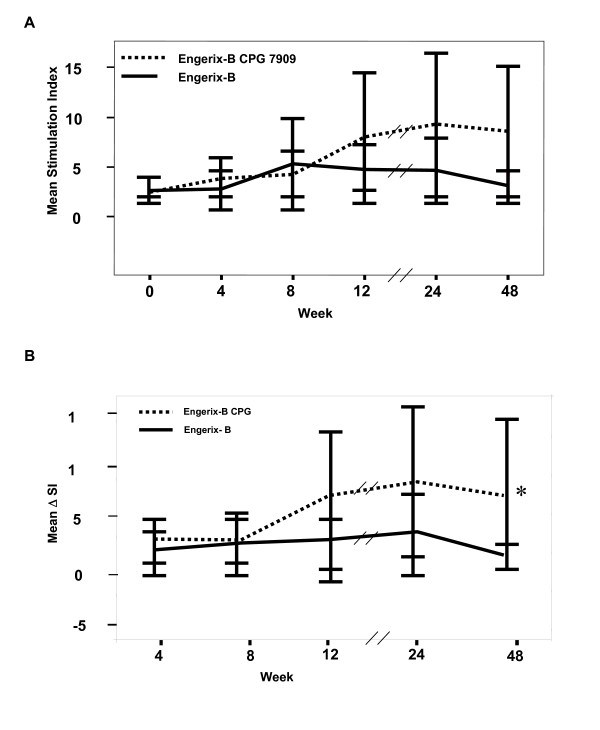
**Proliferative responses to hepatitis B surface antigen (HBsAg)**. A) stimulation index (SI) or B) change in SI from baseline are enhanced over the 48 week study period in subjects that received hepatitis B vaccine plus CpG (solid line; n = 19) as compare to those that received hepatitis B vaccine alone (broken line (n = 19) *p 0.04 by Mann Whitney U Test.

Helper T cell responses over the 48-week study period, as evaluated by lymphoproliferation (expressed as cpm) in response to *ex vivo *stimulation with HBsAg were significantly greater at all time points from 4 weeks onward in subjects receiving CPG 7909 compared to control subjects receiving Engerix-B alone (Figure 1). Proliferative responses were also evaluated by stimulation index (SI), as well as a change in SI from baseline (Figure 2A, B). Consistent with that observed with cpm, the inclusion of CPG 7909 also resulted in greater proliferative responses and greater increases in proliferative response from baseline. Using an SI of 5 at week 48 to define a positive proliferative response to HBsAg, there were 8 responders of 19 subjects (42%) in the group that received CPG 7909 compared to only 3 of 19 (16%) in the control group (p = 0.07 by Chi square test). The mean SI of these responders was 17.3 in the CpG group and 8.3 in the control group.

**Figure 3:**
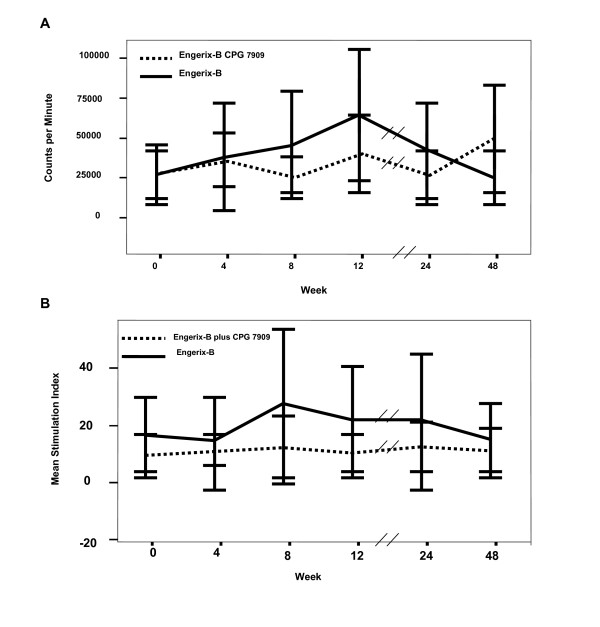
**Proliferative responses to HIV p24 antigen**. Proliferative response to HIV p24 Ag evaluated as A) cpm or B) SI, did not change over the 48 week study period in either subjects that received hepatitis B vaccine plus CpG (solid line; n = 19) or those that received hepatitis B vaccine without CpG (broken line; n = 19).

Proliferative responses were also measured to HIV p24 antigen and other non-vaccine antigens including Candida albicans, CMV and VZV. Whether measured by cpm, SI or change in SI, the inclusion of CPG 7909 had no effect on lymphocyte proliferation to any of these antigens (Figure 3A, B and data not shown).

A post hoc, exploratory analysis was conducted to determine if any correlations existed between cellular immune responses and anti-HBs antibody production. No clear correlation between CpG induced lymphoproliferative responses and absolute anti-HBs antibody titres, seroprotective titres (≥ 10 mIU/ml), or high seroprotective titres (≥ 100 mIU/ml) at week 12 (i.e. one month following administration of all three vaccine doses), week 24 or week 48 was identified.

To further evaluate the impact on host immune function, the effect of CpG on various lymphocyte subsets was also evaluated. There was no change in the proportion of cells that expressed CD4 or CD8, nor was there a change in the proportion of memory CD4 or CD8 cells (CD4CD45RO, CD8CD45RO), naïve CD4 or CD8 cells (CD4CD45RA62L, CD8CD45RA62L), the expression of activation markers on CD4 or CD8 cells (CD4CD38 CD4CD38HLADR, CD8CD38, CD38HLADR) or CD4 or CD8 cells that express either CD28 or CD95 (Fas) (data not shown).

## Discussion

In additional to its ability to improve antibody responses, we demonstrate for the first time in humans that CpG enhances helper T cell responses to vaccine antigen. Moreover, this occurred in a relatively hyporesponsive population. The inclusion of CPG 7909 resulted in enhanced HBV-induced PBMC proliferative responses as measure by a standard ^3^H-thymidine incorporation assay. This was the case whether proliferation was evaluated using raw numbers (cpm) or standardized results (SI), and when it was analyzed as absolute values or as change from baseline, supporting the strength of this observation. The importance of this assay, as compared to newer, perhaps more sophisticated assays, is that it is only enhanced LPA responses that have been shown to predict improved clinical outcomes in a number of human disease states [14,15].

The ability to induce a cellular response to vaccine antigen will be critical for the development of vaccines against pathogens that require such a host response for protection from infection. In fact, it has recently been demonstrated that in a hyporesponsive population, the induction of cellular immune responses, as measured by antigen specific PBMC function, is a better predictor of vaccine induced protection from influenza infection than is antibody response [16]. In the present study, no clear correlation between helper cell responses and antibody production was identified. This may be due to the fact that all subjects that received CpG achieved seroprotective titres, although the variability in the measures of cellular immune response utilized in this study and the relatively small number of participants evaluated may have contributed to this observation.

No enhancement of proliferative responses to mitogen or recall antigen was seen. This suggests that for CpG to enhance an antigen specific immune response, the antigen must be administered along with CpG, perhaps because on the need for physical co-localization. Indeed, mouse studies show that superior results are obtained using alum or liposome-based formulations that provide a depot effect for both the antigen and CpG ODN, compared to immunization with antigen and CpG ODN in saline [5]. No changes were observed in any of the CD4 or CD8 cell subsets, also indicating a lack of general, non-specific effect of CpG on this aspect of the immune system.

## Conclusion

In summary, we have demonstrated that CpG was capable of inducing cellular immune responses to hepatitis B vaccine antigen in effectively treated HIV-infected adults. This has important implications in both improving vaccine responses to currently available vaccines as well as in the development of much needed vaccines where cellular immune responses are thought to be necessary to prevent or treat disease, such as with hepatitis C, HIV and cancer.

## Competing interests

A portion of this work was supported by Coley Pharmaceuticals. HD is an employee of Coley Pharmaceuticals and JBA, CLC and DWC have conducted contract research for Coley Pharmaceuticals.

## Authors' contributions

JBA, CLC, HD and DWC contributed to the design, conduct and analysis of the study. JC was responsible for data analysis. CDY, AC, KGP and ML were responsible for the conduct of the immunological essays involved in the study.
